# Real-world effects of anti-vascular endothelial growth factor injection frequency on visual outcomes in patients with diabetic macular oedema

**DOI:** 10.1038/s41433-024-02998-2

**Published:** 2024-03-06

**Authors:** Carter J. Payne, Urvi Gupta, Christopher M. Maatouk, Blanche L. Kuo, Scott W. Perkins, Rishi P. Singh, Katherine E. Talcott

**Affiliations:** 1 Case Western Reserve University School of Medicine, Cleveland, OH USA; 2 Cleveland Clinic Cole Eye Institute Center for Ophthalmic Bioinformatics, Cleveland, OH USA; 3 Cleveland Clinic Lerner College of Medicine, Cleveland, OH USA

**Keywords:** Outcomes research, Retinal diseases

## Abstract

**Background and objective:**

Anti-vascular endothelial growth factor (VEGF) injections are often administered less frequently in real-world treatment of diabetic macular oedema (DMO) than what was studied in clinical trials. This study aims to characterise real-world DMO treatment patterns and the effect of treatment intervals on patient outcomes.

**Study design/patients and methods:**

This was a retrospective study of 291 patients with DMO treated with anti-VEGF therapy. 12- and 24-month best visual acuity (BVA) and central subfield thickness (CST) were compared between injection interval groups, which were determined by averaging the two most recent injection intervals. Multiple linear regressions were performed to identify factors associated with injection interval, BVA, and CST.

**Results:**

48.8% of patients received injections less than or equal to every 8 weeks (≤ q8w), 27.5% between every 8 to 12 weeks (q8–12w), and 23.7% greater than every 12 weeks (> q12w). Baseline CST was similar (*p* = 0.32), but BVA differed significantly in q8–12w patients (*p* = 0.0095). BVA and CST at 12 months were similar, but q8–12w patients experienced greater 12-month BVA improvement (7.36 ± 12.4 letters) than > q12w patients (1.26 ± 12.3 letters; *p* = 0.0056). 24-month BVA and CST changes were similar between groups (*p* = 0.30 and 0.87). Baseline BVA, HbA1c, and sex were associated with 12-month BVA, and baseline BVA and CST were associated with 12-month CST.

**Conclusion:**

Many patients experienced improvements in BVA and CST over 12 months of treatment despite receiving less frequent anti-VEGF therapy than recommended in the pivotal trials. The present study showed that extended treatment intervals with bevacizumab were effective in preserving vision of many individuals with high baseline BVA.

## Introduction

Diabetic macular oedema (DMO) is a vision-threatening end point of diabetic retinopathy affecting approximately 7% of individuals with diabetes [1]. In the United States, nearly 1 in 25 individuals with diabetes over the age of 40 has DMO in at least one eye [2]. Multiple factors contribute to the pathophysiology of DMO, including a breakdown of the blood-retinal barrier, oxidative stress, and high levels of vascular endothelial growth factor (VEGF) [3]. Ocular treatment of DMO is primarily intraocular pharmacotherapy with anti-VEGF agents and/or steroids.

Anti-VEGF agents, including ranibizumab, aflibercept, and bevacizumab, have surpassed other treatments in their efficacy and are now considered first-line therapy for DMO. The RIDE and RISE phase III clinical trials showed that patients with DMO who received monthly injections of ranibizumab reported larger visual acuity improvements at 24 months when compared to the sham control group [4]. The VIVID and VISTA phase III trials evaluated laser control versus aflibercept regimens of either monthly injections or injections every two months after five initial monthly doses, and both injection groups reported similar efficacy and improved best visual acuity (BVA) over laser after 148 weeks [5]. Finally, bevacizumab injections every six weeks resulted in a larger improvement in gained ETDRS letters when compared to laser therapy over 24 months in the BOLT study [6].

In the aforementioned trials, patients received injections at closely monitored intervals for finite periods of time. In real-world clinical settings, however, a similar consistency in injection frequency is difficult to achieve for a variety of reasons including economic burden with repeated injections, and multiple studies have shown that patients are often undertreated compared to the standards in clinical trials [7–10]. Other studies have examined less stringent dosing regimens, including *pro re nata* (PRN) and treat-and-extend (T&E) regimens, which may allow for reduced treatment burden over the same time period compared to regimens in the previously described clinical trials [11, 12]. The prospective TREX-DMO study of 150 eyes found similar visual acuity outcomes using a T&E regimen when compared to regular monthly injections after 1 year [11]. Despite the promise of trials like TREX- DMO, other studies examining real-world clinical patterns of anti-VEGF treatment have found less improvement in visual outcomes in patients with less frequent injections [13, 14]. The balance between strict monthly regimens and the more flexible T&E and PRN alternatives is still under investigation.

Multiple studies have examined whether patient factors (e.g., baseline BVA, age, systemic metabolic factors) are associated with visual outcomes [15–17]. The association between specific patient factors and injection frequency, however, remains to be explored. Holekamp et al. and the Echo Study Report have suggested specific factors that may contribute to low injection frequency but did not address the topic in their studies [13, 14]. A better understanding of the association between patient factors and injection frequency may help define barriers to effective injection regimens.

This study aims to assess the impact of frequency and timing of anti-VEGF injections on visual and anatomical outcomes in patients with DMO. Additionally, it will characterise real-world anti-VEGF treatment patterns and the real-world patient factors, medical and sociodemographic, that may be significantly related to injection intervals.

## Methods

A retrospective, non-randomised cohort study of patients diagnosed with DMO from January 1, 2012 to December 31, 2019, was performed at Cole Eye Institute, Cleveland OH, after receiving approval from the Cleveland Clinic Investigational Review Board (IRB). All research was performed in adherence to the Declaration of Helsinki. Data was extracted from the electronic medical record system at Cole Eye Institute in Cleveland, OH. All study related procedures were performed in accordance with good clinical practice (International Conference on Harmonisation of Technical Requirements of Pharmaceuticals for Human Use [ICH] E6), applicable FDA regulations, and the Health Insurance Portability and Accountability Act.

Patients who were diagnosed with DMO, over the age of 18, anti-VEGF treatment-naive before diagnosis, and received at least three anti-VEGF injections within one year of anti-VEGF treatment initiation were eligible. Treatment initiation began with a 3-month loading dose and subsequent injections were guided by physician discretion. Treating physicians evaluated patients for the presence of clinically significant oedema and used clinical exams, optical coherence tomography imaging, and vision to drive treatment intervals. Index date was defined as the date of the first anti-VEGF injection. Patients who did not receive an injection within 12 ± 2 months of their index date were excluded, as were those who initiated anti-VEGF outside of our centre, had vitreoretinal surgery at any point prior to or during the treatment period, received steroid injections or implants within four months prior to the index date or at any point during the treatment period, or focal/peripheral laser within three months prior to the index date in the considered eye. Further exclusion criteria included incomplete treatment and past ocular history, the presence of macular oedema for reasons other than DMO (including retinal vein occlusion, age-related macular degeneration, central serous chorioretinopathy, etc.), comorbid ocular conditions that are treated with anti-VEGF agents (including uveitis, neovascular glaucoma, and ocular ischaemic syndrome), pregnancy, and a recent (within six months) history of eye trauma or intraocular surgery, including cataract surgery. If both eyes of the same patient met eligibility criteria, the first eye to receive treatment was the included eye.

Collected data points included basic demographics (gender, race, age at first injection, insurance type, eye laterality), medical factors (HbA1c, serum creatinine, age-adjusted Charlson score for comorbidities, number of inpatient admissions, number of cancelled or missed appointments), and treatment-related variables including index date (date of first injection), injection types and dates of each injection across 24 months, as well as BVA and CST at baseline, 12 months, and 24 months, where available.

The primary aim of the study was to characterise real-world anti-VEGF treatment patterns including mean number of injections, injection type, and injection interval at 12 and 24 months from the index date. The secondary aims were to compare changes in best visual acuity (BVA) and central subfield thickness (CST) in patients with DMO treated with varying intervals of anti- VEGF injection frequency, assess what factors in real-world factors are associated with changes in BVA, and to assess the relationship between patient medical and demographic factors and anti- VEGF injection interval.

Injection interval was measured in weeks and was calculated as the average of the two intervals separating the three injections closest to the 12 ± 2-month injection. This method ensured that the loading doses that are typically administered upon initiation of anti-VEGF therapy (usually comprising three monthly doses, then extension per physician discretion) did not bias the calculation of the patient’s steady-state treatment frequency. Patients were separated into three groups varying by injection interval: less than or equal to 8 weeks (≤ q8w), greater than 8 weeks but less than or equal to 12 weeks (q8–12w), or greater than 12 weeks (> q12w). Additional endpoints included BVA, which was collected by investigators as a Snellen notation and was reported using an Early Treatment Diabetic Retinopathy Study (ETDRS) approximation, and CST, which represented macular thickness and was measured on optical coherence tomography (OCT) images that were received using Cirrus Spectral Domain High Definition-OCT review software.

Demographic and clinical information was summarised with percentages for categorical variables and mean ± standard deviation for continuous variables. ANOVA was used to assess differences in age at baseline between the 12-month injection interval groups. Chi-square tests or Fisher’s exact test when appropriate was used to assess differences in injection medication used, race, laterality, and sex between patients grouped according to 12-month injection interval. Levene’s test followed by ANOVA or Welch’s ANOVA as appropriate were used to determine differences between 12-month injection interval groups with respect to baseline, 12-month, and 24-month BVA and CST. Paired T-tests were used for within-group comparisons of baseline mean BVA and CST to 12- or 24-month BVA and CST. For visual and anatomic outcomes, the analysis was split into two parts. First, 12-month outcomes were assessed for the full 291 patient cohort, with patients grouped according to 12-month injection interval. Second, 12- and 24-month outcomes were assessed for 193 patients with 12- and 24-month BVA and CST data, with patients grouped according to 12-month injection interval. 51 of these 193 patients did not receive an injection within 2 months of their 24-month follow-up, so a 24-month injection interval could be calculated for 142 patients. Finally, multivariable linear regression was performed to assess which baseline factors were associated with a patient’s injection interval as well as 12- and 24-month visual and anatomic outcomes.

## Results

### Demographics

291 eyes were eligible for the study based on the inclusion and exclusion criteria. All 291 patients had follow-up visits at 12 months after treatment initiation; of these, 193 had 24-month follow-up visits, with 142 of those patients receiving injections at 24 ± 2 months. Baseline demographics and clinical characteristics are summarised in Table 1. The mean age was 63.8 ± 9.4 years, and there were slightly more females in the study than males (51.2%). Baseline BVA and CST were 64.3 ± 13.2 ETDRS letters and 423.0 ± 110.8 μm, respectively. A majority of the patients were white (75.2%) and had Medicare insurance (52.9%). The right eye was assessed in 55.7% of patients. 53.6% of patients received only bevacizumab throughout their treatment period, while 3.8% received aflibercept only, 0.3% received ranibizumab only, and 42.3% received a mixed regimen consisting of at least two of the three anti- VEGF agents (37.1% mixed bevacizumab plus aflibercept, 1.0% bevacizumab plus aflibercept plus ranibizumab, and 4.1% mixed bevacizumab plus ranibizumab). Throughout the course of treatment, 13 patients received focal macular laser treatment, nine patients received panretinal photocoagulation laser treatment (PRP), and one patient received both. The average (± standard deviation) HbA1c among all patients was 8.1 ± 1.9%, and the average serum creatinine was 1.35 ± 1.3 mg/dL. On average, patients either missed or cancelled ophthalmology visits 2.8 ± 2.5 times per year.

**Table 1:** Demographics.

Factor	*N*	Statistics
Age, Mean ± SD		63.8 ± 9.4
Baseline BVA (ETDRS), Mean ± SD		64.3 ± 13.2
Baseline CST (μm), Mean ± SD		423.0 ± 110.8
Gender		
Male	149	48.8%
Female	142	51.2%
Race		
White	219	75.2%
Non-white	72	24.7%
Insurance		
Private	85	29.2%
Medicare	154	52.9%
Medicaid	35	12.0%
None	17	5.8%
Eye Laterality		
Right	162	55.7%
Left	129	44.3%
Injection type		
Bevacizumab	156	53.6%
Aflibercept	11	3.8%
Ranibizumab	1	0.3%
Mixed	123	42.3%
Laser treatment^a^		
Focal macular	14	4.8%
PRP	10	3.4%
Diabetes Markers		
HbA1c		8.05 ± 1.9
Serum Creatinine		1.35 ± 1.3
Inpatient admissions per year		0.61 ± 1.32
Missed/cancelled visits per year		2.8 ± 2.5

^a^1 patient received both focal macular and PRP laser treatment and was counted in both groups.

### Injection interval

At 12 months, patients on average received 8.1 ± 2.6 injections. 142 (48.8%) patients were treated ≤ q8w, while 80 (27.5%) were treated q8–12w, and 69 (23.7%) were treated > q12w. Patients in the ≤ q8w group received 9.5 ± 2.3 injections on average during the first year, while those in the > q12w group received an average of 5.9 ± 1.9 injections. When reviewing only patients those with 24-month injection data, the proportion of patients receiving extended treatment intervals, i.e., q8–12w or > q12w, increased from 28.9% to 33.8% and from 19.7% to 27.5% respectively (Table 2). The average number of injections over two years in patients with 24-month data was 14.7 ± 4.1, with 8.53 ± 2.56 injections on average in the first year.

**Table 2:** Injection interval groups and number of injections.

Injections for all patients			
Injection Interval	Anti-VEGF Injections at 12 months for ALL patients		
	*N*	%	Number of injections, Mean ± SD
**≤ 8 weeks**	142	48.8	9.5 ± 2.3
**8–12 weeks**	80	27.5	7.9 ± 1.5
**> 12 weeks**	69	23.7	5.9 ± 1.9
**Overall**	291	100	8.1 ± 2.6

Injections for patients with 24 months follow-up						
Injection Interval	Anti-VEGF Injections at 12 months			Anti-VEGF Injections at 24 months		
	_*N*_	_%_	Number of injections, Mean ± SD	_*N*_	_%_	Number of injections, Mean ± SD
**≤ 8 weeks**	73	51.4	9.78 ± 2.25	55	38.7	17.0 ± 3.5
**8–12 weeks**	41	28.9	8.37 ± 1.70	48	33.8	15.1 ± 3.5
**> 12 weeks**	28	19.7	5.46 ± 1.53	39	27.5	10.9 ± 2.7
**Overall**	142	100	8.53 ± 2.56	142	100	14.7 ± 4.1

Laser treatments by injection interval over the first 12 months were as follows: 6.1%, 2.1%, and 5.3% of patients received focal macular laser in the ≤ q8w, q8–12w, and > q12w groups, respectively; and 1.8%, 3.1%, 7.2% of patients received PRP in the ≤ q8w, q8–12w, and > q12w groups, respectively.

The average age, baseline BVA and CST, sex, race, and laterality amongst the whole cohort are described in Table 3, and there were no significant differences in these measures between the various injection interval groups except for baseline BVA and race. Baseline BVA was 64.2 ± 12.2 letters in the ≤ q8w group, 61.4 ± 16.2 letters in the q8–12w group, and 67.8 ± 10.6 letters in the > q12w group (Table 3). The baseline BVA in the q8–12w group was significantly lower than that of the > q12w group (*p* = 0.008). The q8–12w group additionally had a significantly higher proportion of white individuals than both the ≤ q8w group (*p* = 0.02) and > q12w group (*p* = 0.02). A Fisher’s exact test showed no difference between groups with respect to distribution of insurance types (*p* = 0.67). A significant difference was found between the three groups with respect to the type of anti-VEGF agents received, with the q12w group having the highest frequency of bevacizumab-only and lowest frequency of mixed regimen (*p* < 0.001).

**Table 3:** Baseline demographic descriptive statistics by injection interval.

Variable	Level	All (*n* = 291)	≤ q8w	q8–12w	> q12	*p*-value
Age, Mean ± SD		63.8 ± 9.4	63.9 ± 8.8	64.6 ± 9.6	62.7 ± 10.2	0.49
Baseline BVA, Mean ± SD		64.3 ± 13.2	64.2 ± 12.2	61.4 ± 16.2	67.8 ± 10.6	0.027
Baseline CST, Mean ± SD		423.0 ± 110.8	420.2 ± 99.8	429.8 ± 123	420.8 ± 118	0.84
Sex, *N* (%)	Male	149 (48.8)	70 (49.3)	45 (56.3)	34 (49.3)	0.57
	Female	142 (51.2)	72 (50.7)	35 (43.7)	35 (50.7)	
Race, *N* (%)	White	219 (75.2)	102 (71.8)	70 (87.5)	47 (68.1)	0.009
	Non-white	72 (24.7)	40 (28.2)	10 (12.5)	22 (31.9)	
Affected Eye, *N* (%)	Right	162 (55.7)	75 (52.8)	45 (56.3)	42 (60.9)	0.54
	Left	129 (44.3)	67 (47.2)	35 (43.7)	27 (39.1)	
Injection medication	Bevacizumab	156 (53.6)	60 (42.3)	46 (57.5)	50 (72.5)	2.1e–06
	Ranibizumab	1 (0.3)	1 (0.7)	0	0	
	Aflibercept	11 (3.8)	2 (1.4)	3 (3.8)	6 (8.7)	
	Mixed Regimen	123 (42.3)	79 (55.6)	31 (38.8)	13 (18.8)	

Several predictors including various demographic and medical characteristics were assessed with multiple regression analysis to predict the injection interval at 12 and 24 months. None of the predictors were significantly correlated with a specific injection interval for the 12- month data. For the 24-month data, the number of inpatient hospital admissions was shown to be significantly predictive of injection interval (*p* = 0.03) and had an effect size of + 8.3 weeks/injection (Table S1). The average numbers of inpatient admissions for the 24-month data were 0.44, 0.47, and 1.01 admissions for the < q8wks, q8–12, and > q12wks injection interval groups, respectively.

### Visual acuity outcomes

**Fig. 1:**
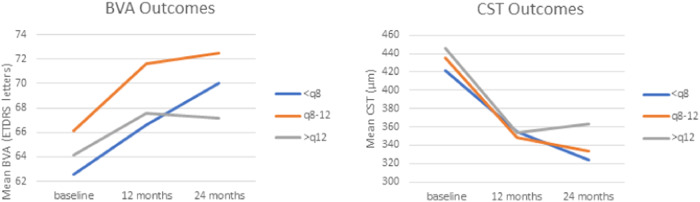
Outcomes of patients undergoing injections for diabetic macular edema, including best-corrected visual acuity (BVA) and central subfield thickness (CST) for patients over 24 months, stratified by injection frequency.

At 12 months, BVA was 68.7 ± 12.2 letters in the ≤ q8w group, 69.3 ± 10.2 letters in the q8–12w group, and 69.0 ± 12.6 letters in the > q12w group, with no significant difference between the three (*p* = 0.95) (Table 4, Fig. 1). However, the change in BVA from baseline to 12 months was significantly higher in the q8–12w group at 7.36 ± 12.4 letters than in the > q12w group at 1.26 ± 12.3 letters (*p* = 0.0056). The 12-month change in BVA was 4.07 ± 12.0 letters in the ≤ q8w; this was not significantly different from the 12-month BVA change in either the > q12w or q8–12w groups (*p* = 0.127 and 0.083, respectively). The change in BVA from baseline to 12 months was significant in the ≤ q8w group and q8–12w groups (*p* < 0.001), but not in the > q12w group (*p* = 0.44).

**Table 4:** Patient outcomes at baseline and 12-months by 12-month injection interval subgroups.

Factor	*N*	≤ q8w	*N*	q8–12w	*N*	> q12	*p*-value^1^
**BVA (ETDRS), Mean ± SD**							
Baseline	142	64.2 ± 12.2	80	61.4 ± 16.2	69	67.8 ± 10.6	0.027
12 Month	142	68.7 ± 12.2	80	69.2 ± 10.2	69	69.0 ± 12.6	0.94
Change from baseline	142	4.35 ± 11.6	80	7.34 ± 12.4	69	1.26 ± 12.3	0.048
*p*-value^2^ (Baseline vs. 12 month)		1.76e–05		1.32e–06		0.40	
**CST (μm), Mean ± SD**							
Baseline	142	420 ± 100	80	429 ± 123	69	420 ± 118	0.84
12 Month	142	345 ± 91	80	341 ± 87	69	363 ± 103	0.54
Change from baseline	142	−75.6 ± 104	80	-87.9 ± 125	69	−59.4 ± 129	0.28
*p*-value^2^ (Baseline vs. 12 month)		1.47e–14		2.21e–08		0.00036

When analysing only 193 patients with 12- and 24-month BVA and CST values, similar trends were noted with respect to 12-month outcomes (Table S2). 24-month BVA was similar between the three 12-month injection interval groups (*p* = 0.73); however, the change in BVA from baseline to 24 months was again higher in the q8–12w group than in the > q12w group (9.3 ± 15.4 letters vs. 2.0 ± 12.3 letters; *p* = 0.02). However, baseline BVA was again significantly lower in the q8–12w group than in the > q12w group (60.5 ± 16.5 vs. 69.3 ± 9.32 letters; *p* = 0.005). BVA for the groups at 24 months was as follows: 70.0 ± 10.0 for the ≤ q8w group, 69.8 ± 10.0 for the q8–12w group, and 71.4 ± 8.8 letters for the > q12w group. BVA at 24 months did change significantly from baseline in the ≤ q8w and q8–12w groups (*p* < 0.001), though not in the > q12w group (Table S2).

Multiple linear regression analysis was performed to assess which baseline factors can be used to predict 12- and 24-month visual acuity outcomes. Of all the factors measured in Table S2, sex, HbA1c, and baseline BVA were significant predictors of 12-month BVA with an effect size of 0.94, −0.19, and 0.13, respectively (*p* < 0.05). There were no significant predictors of 24-month BVA.

### CST outcomes

The baseline CST across the injection interval groups was similar: 420 ± 100 μm for the ≤ q8w group, 429 ± 123 μm for the q8–12 group, and 420 ± 118 μm for the > q12 group (Table 4, Fig. 1). There was no significant difference between the 12-month CST values across the groups or in the change from baseline to 12 months. Within each interval group, there was a significant difference between the baseline and 12-month CST values for all groups (*p* < 0.05). The ≤ q8w group had a change of −69.7 ± 115 μm between baseline and 12 months, while the q8–12w group had a change of −83.0 ± 132 μm, and the > q12w group had a change of −45.7 ± 152 μm. To further explore whether the lack of statistical difference in CST change between injection interval groups may have been influenced by outliers, patients were classified according to which quartile of 12-month CST change they fell in. Chi square analysis comparing distribution of patients within each injection interval group among CST quartiles demonstrated no significant difference between the three injection interval groups (*p* = 0.53).

Similar trends were observed for 12-month CST outcomes when analysing only the 193 patients with 24-month CST data (Table S2). The CST values at 24 months were similar between the 12-month injection interval groups (*p* = 0.19), as were the changes in CST from baseline to 24 months (*p* = 0.32). CST values for the groups at 24 months were as follows: 322 ± 94.5 μm for the ≤ q8w group, 324 ± 82.1 μm for the q8–12w group, and 353 ± 91.4 μm for the > q12w group. CST at 24 months changed significantly from baseline for all groups (*p* < 0.05) (Table S2).

Table S3 shows the results of a multiple linear regression analysis to assess which baseline factors are predictive of 12- and 24-month CST outcomes. Of all the factors measured in Table S3, race, baseline BVA, and baseline CST were significant predictors of 12-month CST with an effect size of -27, 1.1, and 0.25, respectively (*p* < 0.05). Race was also a significant predictor of CST at 24 months, with an effect size of −48 (*p* = 0.03).

To further assess whether injection interval is related to 12- and 24-month CST and BVA change while holding anti-VEGF agent, baseline BVA, and baseline CST constant, an additional regression analysis was conducted using these factors. Baseline BVA was found to be the only significant correlate with 12- and 24-month BVA change. Both baseline BVA and CST were significantly correlated with 12-month CST change, and baseline CST was the only significant correlate with 24-month CST change. Injection regimen and anti-VEGF agent were not significantly correlated with 12- and 24-month outcomes in this analysis.

## Discussion

While anti-VEGF injections have been shown to be effective in improving BVA and reducing oedema in DMO patients through several clinical trials, these trials have relied upon monthly to bimonthly injection intervals which are often not adhered to in real-world settings [7–10]. Several studies have been conducted exploring the real-world outcomes of DMO patients receiving anti-VEGF therapy, though this represents one of few to characterise injection frequencies and their effect on BVA and CST. Additionally, this study is unique in its exploration of the factors which may influence the frequency with which patients are treated for DMO in a real-world setting.

48.8% of patients in our study were treated ≤ q8w during the first 12 months of follow-up, meaning that a majority of patients received treatments less frequently than recommended by the pivotal clinical trials. This is similar to several previous studies, which reported that on average patients received from 2.6 to 6.8 anti-VEGF injections in their first year of follow-up [7–10, 14, 18, 19], At baseline, the three treatment frequency groups were similar in demographic values including age, sex, and baseline CST. Interestingly, the only variable in which the groups differed was baseline BVA and race; the > q12w group had the highest baseline BVA, while the q8–12w group had the lowest (Table 3). The difference in baseline BVA was significant between the > q12w group and q8–12w group, which may suggest that providers are likely to treat patients with worse baseline vision more frequently. Of note, no association was identified between baseline BVA and injection frequency in our multiple linear regression. The q8–12w group additionally had a higher proportion of white individuals than in the ≤ q8w group (*p* = 0.02) and > q12w group (*p* = 0.02). It is therefore possible that the different outcomes observed may have been impacted by the racial breakdown of the injection interval groups. This seems a less likely contributor than baseline BVA, however, given the findings of the multivariable regression analysis (Table S3). It is also interesting to note that the q12w group had the highest frequency of bevacizumab-only injection regimens and lowest frequency of mixed injection regimens, despite the fact that aflibercept has demonstrated efficacy at longer intervals than bevacizumab or ranibizumab, and one might expect the longer interval group to have a higher proportion of aflibercept regimens. Further investigation into the reasons for this unexpected finding are warranted.

The BVA at 12 months was similar between the three injection interval groups; however, the change in BVA from baseline to 12 months differed significantly between groups with the q8–12w group experiencing the greatest 12-month BVA improvement. The difference in 12-month BVA improvement between the q8–12w group and > q12w group was significant. The same trend was noted with respect to 12- and 24-month BVA outcomes when only patients with 24-month BVA data were analysed (Table S2). Though this may suggest that higher injection frequency improves vision outcomes, the significantly better BVA improvement is more likely secondary to the lower baseline VA in the q8–12w group as there was no significant difference in 12-month BVA change between the ≤ q8w group and the q8–12w group. This is further supported by our multiple linear regression analysis, which identified baseline BVA as a significant predictor of 12-month BVA.

Additionally, the distribution of injection frequencies shifted from the first year of treatment to the second, with the proportion of patients receiving injections q8–12w or > q12w increasing (Table 2). Despite this, patients maintained their improvements in visual acuity from the first year of treatment and CST continued to decline (Table 4 and S2). Taken together, this data suggests that individuals with DMO may be able to achieve similar anatomic and visual outcomes after 12 and 24 months of treatment despite q8–12w treatment intervals and potentially even > q12w in individuals with high baseline BVA, both of which are substantially longer than those studied in the pivotal clinical trials of anti-VEGF treatment for DMO. Additionally, patients can maintain their improvements over a second year of treatment despite less frequent injections on average. However, the difference in baseline BVA among the groups in our study suggests that individuals with lower baseline BVA may require more frequent injections to achieve these results.

We also note that there was a greater change in CST relative to change in BVA across groups in our study. This apparent discordance between degree of improvement in anatomy versus improvement in visual function has been reported in other studies. Bressler et al. (2019) reported no significant association between change in CST and change in visual acuity in patients treated for DMO with anti-VEGF injections, similar to our results [20].

In regard to what personal patient factors may influence injection frequency, our analysis found no significant relationship between average injection interval and sociodemographic or medical factors. The number of inpatient admissions a patient underwent was the only significant predictor of injection interval over 24 months, and this is likely secondary to treatment interruption due to illness or hospitalisation, with the > q12w group having on average more than double the number of inpatient admissions compared to the other injection groups. Future studies examining specific reasons for inpatient admission and their contribution to injection interval are warranted. No factors were significantly associated with the injection interval over 12 months. This analysis suggests that a patient’s individual background or medical context has little influence on the frequency at which they receive anti-VEGF injections.

Our study does carry several limitations. Given the nature of anti-VEGF treatment in real-world settings, which frequently involves a 3-month loading dose followed by extension of injection intervals, it is difficult to calculate a precise treatment frequency over the first year of treatment. To address this, our study calculated the average of the intervals between the two most recent injections prior to the 12-month visit, as was done in previous studies of real-world anti- VEGF treatment patterns [21, 22]. Additionally, while all patients had data through at least 12-months, a significantly smaller proportion had data for the full 24-month period, potentially introducing bias in our 24-month results. The attrition rate in this study was relatively high at 33.7%, and most studies on anti-VEGF treatment of DMO do not record data beyond 12 months making it difficult to compare our rate to other real-world reports, however we do note even higher attrition rates from 12 to 24 months have been reported in treatment of DMO (71% reported by Blinder et al., 2017) and in treatment of neovascular age-related macular degeneration with anti-VEGF injections (58% for ranibizumab, 64% for aflibercept reported by Kiss et al., 2020) [8, 14]. All patients were recruited from a single tertiary-centre in Cleveland, Ohio, meaning that our results may not be generalisable to all patient populations. Also, HbA1c values were only available for all patients at their initial treatment visit, but were not available or collected for subsequent visits, limiting analysis of this point. Finally, patients who received steroid injections for treatment of DMO were excluded, further limiting generalisability of this study’s results.

This retrospective study of 291 patients treated with anti-VEGF injections for DMO examined the treatment frequencies of DMO patients in a real-world setting, factors associated with treatment frequency, and how treatment frequency can impact visual acuity and CST outcomes. Our results suggest that patients with higher baseline BVA may achieve similar 12- and 24-month BVA and CST despite less frequent injection treatments. Furthermore, patients are able to maintain their 12-month improvements over 24 months despite fewer injections on average during the second year. This suggests that for a large portion of patients, particularly those with higher baseline BVA who receive a higher relative proportion of bevacizumab treatments, monthly to bimonthly injections may be more frequent than necessary to maintain desirable vision. Extending injection intervals in these patients, particularly after the first year of treatment, may be an effective way to reduce the treatment burden of anti-VEGF therapy on patients.

## Summary

### What was known before

- Anti-vascular endothelial growth factor therapy has been shown to be effective in numerous clinical trials for the treatment of clinically significant diabetic macular oedema.
- However, participants in pivotal clinical trials were treated every 4 or every 8 weeks with injections. Several studies have found that patients do not receive injections this frequently in real-world settings.

### What this study adds

- This study characterises the real-world treatment patterns of patients with diabetic macular oedema seen in a tertiary care centre.
- Additionally, this is the first study to compare visual and anatomic outcomes between patients receiving treatment at different intervals and utilised regression analysis to identify factors associated with treatment intervals and visual/anatomic outcomes.

### Supplementary information

Table S1

Table S2

Table S3

Table S4

Table S5

## Data Availability

The datasets generated during and/or analysed during the current study are available from the corresponding author on reasonable request.
