# Supplementary material for: Real-world effects of anti-vascular endothelial growth factor injection frequency on visual outcomes in patients with diabetic macular oedema
Source: Eye (Lond). 2024 Mar 6;38(9):1687–93. doi: 10.1038/s41433-024-02998-2 (PMC11156885; doi:10.1038/s41433-024-02998-2)
Supplement: Supplementary file 1 — Table S1 [file 41433_2024_2998_MOESM1_ESM.pdf]

Table S1. Patient factors as predictors of injection interval at 12 months and 24 months

Variable	12 Months		24 Months	
	Effect Size	p-value	Effect Size	p-value
Age	-0.40	0.06	0.51	0.21
Sex	0.82	0.83	-1.8	0.79
Race	5.9	0.17	8.1	0.32
Affected Eye	2.0	0.61	-6.5	0.34
A1C	1.1	0.27	-1.7	0.29
Serum creatinine	3.0	0.07	-1.7	0.69
Inpatient visits	-2.3	0.09	8.3	**0.03**
Cancelled visits	-0.63	0.44	-0.30	0.85
Baseline BVA	-0.091	0.54	0.12	0.73
Baseline CST	0.023	0.18	-0.032	0.58
