# Supplementary material for: Real-world effects of anti-vascular endothelial growth factor injection frequency on visual outcomes in patients with diabetic macular oedema
Source: Eye (Lond). 2024 Mar 6;38(9):1687–93. doi: 10.1038/s41433-024-02998-2 (PMC11156885; doi:10.1038/s41433-024-02998-2)
Supplement: Supplementary file 2 — Table S2 [file 41433_2024_2998_MOESM2_ESM.pdf]

Table S2. Patient outcomes at baseline, 12-months, and 24-months by 12-month injection interval subgroups using only patients with 24 months of follow-up data.

Factor	N	≤ q8w	N	q8-12w	N	>q12	p-value[1]
**BVA (ETDRS), Mean ± SD**							
Baseline	101	65.1±11.5	54	60.5±16.5	38	69.3±9.32	**0.020**
. 12 Month	101	68.8±12.1	54	68.9±10.4	38	70.9±8.4	0.83
Change from baseline	101	3.3±11.4	54	8.4±13.0	38	1.5±9.5	**0.037**
p-value[2] (Baseline vs. 12 month)		**0.004**		**1.74e-05**		0.32	
. 24 Month	101	70.9±10.0	54	69.8±10.0	38	71.4±8.8	0.70
Change from baseline	101	5.4±11.7	54	9.3±15.4	38	2.0±12.3	0.10
p-value[2] (Baseline vs. 24 month)		**1.46e-05**		**4.69e-05**		0.32	
**CST (µm), Mean ± SD**							
Baseline	101	414±98.5	54	446±122	38	436±118	0.30
. 12 Month	101	341±90.3	54	347±81.4	38	361±101	0.68
Change from baseline	101	-73.1±110	54	-98.8±128	38	-72.8±141	0.51
p-value[2] (Baseline vs. 12 month)		**1.76e-09**		**5.88e-07**		**3.28e-03**	
. 24 Month	101	322±94.5	54	324±82.1	38	353±91.4	0.11
Change from baseline	101	-91.3±117	54	-122±142	38	-85.1±163	0.43
p-value[2] (Baseline vs. 24 month)		**1.13e-11**		**5.88e-08**		**0.0031**	
