# Supplementary material for: Real-world effects of anti-vascular endothelial growth factor injection frequency on visual outcomes in patients with diabetic macular oedema
Source: Eye (Lond). 2024 Mar 6;38(9):1687–93. doi: 10.1038/s41433-024-02998-2 (PMC11156885; doi:10.1038/s41433-024-02998-2)
Supplement: Supplementary file 3 — Table S3 [file 41433_2024_2998_MOESM3_ESM.pdf]

Table S3: Distribution of Patient within each Quartile of CST Variability among Injection Interval Groups

	Percent of Patients in each Injection Interval Group			
**Quartile of 12-month CST Change**	<q8w	q8-12w	>q12w	**Total Patients**
First	46%	33%	21%	72
Second	56%	25%	18%	71
Third	51%	23%	27%	71
Fourth	43%	29%	28%	72
**Chi-Square Test P-value: 0.53**				
