# Supplementary material for: Real-world effects of anti-vascular endothelial growth factor injection frequency on visual outcomes in patients with diabetic macular oedema
Source: Eye (Lond). 2024 Mar 6;38(9):1687–93. doi: 10.1038/s41433-024-02998-2 (PMC11156885; doi:10.1038/s41433-024-02998-2)
Supplement: Supplementary file 4 — Table S4 [file 41433_2024_2998_MOESM4_ESM.pdf]

Table S4. Patient factors as predictors of change in BVA and CST

	**12 Months**				**24 months**			
**Variable**	**BVA Effect size**	**p-value**	**CST Effect size**	**p-value**	**BVA Effect size**	**p-value**	**CST Effect size**	**p-value**
Age	0.0035	0.85	0.13	0.83	-0.020	0.48	0.081	0.94
Sex	0.94	**0.0049**	4.2	0.71	0.12	0.81	3.9	0.83
Race	-0.17	0.64	-27	**0.030**	0.35	0.54	-48	**0.030**
Affected Eye	-0.44	0.17	-4.8	0.67	0.25	0.60	23	0.22
A1C	-0.19	**0.030**	4.7	0.12	-0.019	0.87	3.8	0.39
Serum creatinine	0.032	0.82	-5.9	0.23	0.049	0.87	-3.2	0.78
Inpatient visits	-0.077	0.51	2.5	0.53	0.27	0.31	12	0.24
Cancelled visits	-0.014	0.84	3.3	0.17	-0.052	0.64	-6.2	0.15
Baseline BVA	0.13	**2.0e-16**	1.1	**0.010**	0.037	0.14	0.67	0.49
Baseline CST	0.0022	0.14	0.25	**2.1e-6**	0.0028	0.24	0.075	0.42
