# Supplementary material for: Real-world effects of anti-vascular endothelial growth factor injection frequency on visual outcomes in patients with diabetic macular oedema
Source: Eye (Lond). 2024 Mar 6;38(9):1687–93. doi: 10.1038/s41433-024-02998-2 (PMC11156885; doi:10.1038/s41433-024-02998-2)
Supplement: Supplementary file 5 — Table S5 [file 41433_2024_2998_MOESM5_ESM.pdf]

Table S5. Multiple linear regression analyzing relation between interval groups and injection regimens and 12- and 24-month BVA and CST change.

Factor	12-month BVA Change		12-month CST Change		24-month BVA Change		24-month CST Change	
	Estimate	P-value	Estimate	P-value	Estimate	P-value	Estimate	P-value
Intercept	34.45	**<0.001**	134.35	**0.00**	56.34	**<0.001**	206.91	**0.00**
>q12w Group	0.07	0.96	14.35	0.29	-0.32	0.87	27.13	0.15
q8-12w Group	2.21	0.13	-2.57	0.83	0.58	0.72	1.79	0.91
<q8w Group	Reference Level							
Baseline BVA	0.48	**<0.001**	1.02	**0.01**	-0.72	**<0.001**	0.84	0.11
Baseline CST	0.00	0.56	-0.68	**<0.001**	0.00	0.54	-0.90	**<0.001**
Bevacizumab Only Regimen	0.60	0.85	11.00	0.69	-2.48	0.50	13.85	0.70
Mixed Regimen	2.43	0.46	14.95	0.60	-1.47	0.70	27.61	0.45
Ranibizumab Only Regimen	13.40	0.21	-61.93	0.50	-18.35	0.07	-100.54	0.31
Aflibercept Only Regimen	Reference Level							
Adjusted R-squared	0.27	**<0.001**	0.45	**<0.001**	0.51	**<0.001**	0.55	**<0.001**
